# Supplementary material for: Locus Coeruleus tracking of prediction errors optimises cognitive flexibility: An Active Inference model
Source: PLoS Comput Biol. 2019 Jan 4;15(1):e1006267. doi: 10.1371/journal.pcbi.1006267 (PMC6334975; doi:10.1371/journal.pcbi.1006267)
Supplement: S1 Appendix — (PDF) [file pcbi.1006267.s001.pdf]

Derivations for Active Inference update equations, state-action prediction errors and model decay

The following provides a condensed derivation of the Active Inference expressions in Boxes 1 and 2 (as originally described by [4, 3]), together with an account of the extension of the theory to include state-action prediction errors and model decay factors.

1 Free Energy

If o represents an observation (a sensory input), and x represents hidden states and parameters in a model i.e. different possible causes of the sensory input, then the probability of different causes of sensory stimuli can be described by the posterior distribution

$$P(x \mid o) = \frac{P(o \mid x)P(x)}{P(o)}.$$

When the probability distributions are simple enough, inferences can be made by computing this posterior directly. If this is not the case, a possible way forward is to propose an approximate version of $P(x \mid o)$ and optimise its sufficient statistics [5, 1, 2]. If $Q(x)$ represents the approximate distribution, then the Kullbeck Liebeck divergence between the true and approximate posterior distributions can be written

$$D_{KL}(Q \parallel P) = -\int dx\ Q(x)\ ln \left[\frac{P(x \mid o)}{Q(x)}\right].$$

Since $P(o,\, x) = P(x \mid o)\, P(o)$,

$$\begin{aligned} D_{KL}(Q \parallel P) &= -\int dx\ Q(x)\ \left[ln \left[\frac{P(o,\, x)}{P(o)}\right] - ln\, Q(x)\right] \\ &= \int dx\ Q(x)\ ln\, P(o) - \int dx\ Q(x)\ ln \left[\frac{P(o,\, x)}{Q(x)}\right] \\ &= ln\, P(o) + F \end{aligned}$$

The first term in this expression is the log of the model evidence for the observations under P. This term is a constant (it does not rely on Q). To minimize the distance between P and Q, we therefore focus on minimising F, the "variational free energy":

$$F = -\int dx\ Q(x)\ ln \left[\frac{P(o,\, x)}{Q(x)}\right] = E_{Q(x)} \left[ln\, (Q(x) - ln\, P(o, x)\right] \tag{1}$$

Where the $E_{Q(x)}$ represents the expected value under $Q(x)$. Since $P(o, x) = P(o \mid x)\, P(x)$, this can also be written in the form

$$F = D_{KL}\left[Q(x) \parallel P(x)\right] - E_{Q(x)}\left[ln\, P(o \mid x)\right]. \tag{2}$$

2 Generative model \ approximate posterior

Under Active Inference, the agent's generative model is a Partially Observed Markov Decision Process (POMDP) with joint probability over observations $\tilde{o}$ and causes of those observations $x = (\tilde{s}, a, b, d, \pi, \gamma)$:

$$P(\tilde{\mathrm{o}}, \tilde{\mathrm{s}}, \pi, a, b, d, \beta) = P(\boldsymbol{\pi})\, P(\boldsymbol{A})\, P(\boldsymbol{B})\, P(\boldsymbol{D})\, P(\beta) \prod_{t=1}^{T} P(o_t \mid s_t)\, P(s_t \mid s_t - 1, \pi). \tag{3}$$

Where $\tilde{\boldsymbol{o}}$ and $\tilde{\boldsymbol{s}}$ represent sequences of probabilities of observations and states over time from $t = 0$ to the current time (whilst $\boldsymbol{o}$ and $\boldsymbol{s}$ would represent the probability distributions of observations and states at a single time). Note that the causes of observations in the model include both unknowns associated with the task (a, b, d) and unknown effects which arise as a result of the agent's own actions (π, γ). As described in Box 2:

- $\boldsymbol{A}$ is a matrix representing the probability of specific outcomes being observed from specific states such that $P(o_t^i \mid s_t^j) = \boldsymbol{A_{ij}}$. Each column of $\boldsymbol{A}$ is a categorical distribution providing the probability of each outcome arising from a single state. The columns of $\boldsymbol{A}$ are Dirichlet distributions parameterised by the concentration parameters in the corresponding column of the matrix $\boldsymbol{a}$ such that $P(\boldsymbol{A}) = Dir\,(\boldsymbol{a})$.
- $\boldsymbol{B(u)}$ describes the probabilities of state transitions under action u, such that each column of $\boldsymbol{B}$ is a categorical distribution providing the probabilities of states at time $t+1$, given a starting state at time t and an action u: $P(s_{t+1} \mid s_t) = \boldsymbol{B}$; $P(\boldsymbol{B}(u)) = Dir\,(\boldsymbol{b}(u))$.
- $\boldsymbol{D}$ describes beliefs about the state occupied at t=1: $P(s_1 \mid s_0) = \boldsymbol{D}$; $P(\boldsymbol{D}) = Dir\,(\boldsymbol{d})$
- The precision parameter γ , which describes confidence in beliefs (see [3]) is a gamma distribution such that $P(\gamma) = \Gamma(\alpha, \beta)$. Here, $\alpha = 1$ throughout.
- Each policy is a sequence of actions over time. The vector $\boldsymbol{\pi}$ holds the probabilities of individual policies. These probabilities depend on the free energy expected in the future under a specific policies $(G(\pi))$, given current beliefs:
$$ln\,[P(\boldsymbol{\pi})] \propto -\gamma\, G(\pi)\,, \quad P(\boldsymbol{\pi}) = \sigma(-\gamma\, G(\pi)),$$
where σ denotes the softmax function (which normalises the probabilities, e.g. $\sigma(\,-\gamma.\cdot G(\pi_j)\,) = \dfrac{e^{-\gamma \cdot G(\pi_j)}}{\sum_i e^{-\gamma \cdot G(\pi_i)}}$).
Note that this is an additional requirement within the model to minimise free energy: the entire parameter space is chosen to minimise free energy, and within that constraint, individual policies are more probable if they have a lower expected free energy.

The agent's approximate posterior over hidden states and parameters is assumed to have a similar form:

$$Q(\tilde{\boldsymbol{s}}, \boldsymbol{\pi}, \boldsymbol{a}, \boldsymbol{b}, \boldsymbol{d}, \boldsymbol{\beta}) = Q(\pi) Q(A) Q(B) Q(D) Q(\beta) \prod_{t=1}^{T} Q(s_t \mid \pi) \tag{4}$$

Note that the distributions in the approximate posterior for states, policies and parameters are expressed in terms of their sufficient statistics (their expected values), and are denoted in bold: $\boldsymbol{a}, \boldsymbol{b}, \boldsymbol{d}, \boldsymbol{\gamma}, \boldsymbol{\pi}, \boldsymbol{s}$. Finally, in keeping with the derivations in [4, 3], we use the dot product $\boldsymbol{A} \cdot \boldsymbol{B} = \boldsymbol{A^T B}$ for both vectors and matrices.

3 Variational free energy and update equations

Equations (3) and (4) can then be used in (1) to yield the following expression for free energy:

$$F = E_{Q(x)}\left[ln\left(Q(\pi)Q(A)Q(B)Q(D)Q(\beta)\prod_{\tau=1}^{T}Q(s_\tau \mid s_{t-1},\pi)\right) - ln\left(P(\boldsymbol{\pi})\,P(A)\,P(B)\,P(D)\,P(\beta)\prod_{t=1}^{T}P(o_t \mid s_t)\,P(s_t \mid s_t-1,\pi)\right)\right] \tag{5}$$

$$F = D_{KL}\left[Q(\pi) \parallel P(\pi)\right] + D_{KL}\left[Q(A,B,D,\gamma) \parallel P(A,B,D,\gamma)\right] + E_{Q(x)}\left[\sum_{\tau=1}^{T}\left(ln\frac{Q(s_\tau \mid \pi)}{P(s_\tau \mid s_{\tau-1},\pi)} - ln\,P(o_\tau \mid s_\tau)\right)\right] \tag{6}$$

In order to derive updates which minimise free energy, this expression will be re-expressed in terms of model parameters, then differentiated with respect to each individual parameter and set to zero. We will begin by deriving the update equation for states.

3.1 The variational free energy of states under different policies ($F(\pi)$)

The third term of the equation (6) describes the dependence of the variational free energy F on states (s). Comparing with equation (2), it is clear that this term is itself the sum over time points of more specific free energies - those of states when a specific policy is being followed. This can be written:

$$\begin{aligned}
F(\pi) &= \sum_{\tau=1}^{T}F(\pi,\tau)\\
&= E_{Q(s_\tau,o_\tau|\pi)}\left[\sum_{\tau=1}^{T}ln\frac{Q(s_\tau \mid \pi)}{P(s_\tau \mid s_{\tau-1},\pi)} - ln\,P(o_\tau \mid s_\tau)\right]\\
&= \sum_{\tau=1}^{T}\boldsymbol{s^\pi_\tau}\cdot\left[ln(\boldsymbol{s^\pi_\tau}) - ln\,(\boldsymbol{B^\pi_{\tau-1}})\boldsymbol{s^\pi_{\tau-1}} - ln(\boldsymbol{A})\cdot\boldsymbol{o}_\tau\right]
\end{aligned}$$

Where the agent's belief about the current state at time τ under a given policy - $Q(s_\tau \mid \pi)$ - has been re-written simply as $\boldsymbol{s}^\pi_\tau$. The probability of a state transition under a given policy, $P(s_\tau \mid s_{\tau-1},\pi)$, is equal to $\boldsymbol{B}^\pi_{\tau-1}$ (that is, the $\boldsymbol{B}$ matrix for the action prescribed by policy π at time $\tau-1$). Finally, expectation values have been rewritten using dot products (e.g. $E(x) = \sum_x P(x)\,x = \boldsymbol{P}\cdot\boldsymbol{x}$ if $\boldsymbol{P}$ is a vector of probabilities for different values of x held in the vector $\boldsymbol{x}$).

Note that at $\tau = 1$ the probability of transitioning from a 'previous state' (given by a $\boldsymbol{B}$ matrix) is replaced by the initial probability of states, $\boldsymbol{D}$:

$$F(\pi,1) = \boldsymbol{s^\pi_1}\cdot\left[ln(\boldsymbol{s^\pi_1}) - ln\,\boldsymbol{D} - ln(\boldsymbol{A})\cdot\boldsymbol{o}^\pi_1\right]. \tag{7}$$

This will be useful when deriving the update equation for D below. The dependence of the free energy on states (the third term of equation (6)) can then be written simply as

$$E_{Q(\pi)}\left[\boldsymbol{F_\pi}\right] = \boldsymbol{\pi}\cdot\boldsymbol{F_\pi}$$

Where $\boldsymbol{F_\pi} = (F(\pi_1), F(\pi_2)...)$ and $\boldsymbol{\pi}$ is the vector holding the probability of each policy.

3.2 Update equations for states (fast updates)

Differentiating the expression for free energy with respect to states, and setting the result to zero:

$$\frac{\partial F}{\partial \boldsymbol{s}^{\boldsymbol{\pi}}_{\boldsymbol{\tau}}} = \frac{\partial F}{\partial F_{\pi}}\frac{\partial F_{\pi}}{\partial \boldsymbol{s}^{\boldsymbol{\pi}}_{\boldsymbol{\tau}}} = \boldsymbol{\pi} \cdot \left[ln\,(\boldsymbol{s}^{\boldsymbol{\pi}}_{\boldsymbol{\tau}}) - ln\,(\boldsymbol{B}^{\boldsymbol{\pi}}_{\boldsymbol{\tau-1}})\boldsymbol{s}^{\boldsymbol{\pi}}_{\boldsymbol{\tau-1}} - ln(\boldsymbol{A}) \cdot \boldsymbol{o}^{\pi}_{\tau} + ln\,(\boldsymbol{B}^{\boldsymbol{\pi}}_{\boldsymbol{\tau}}) \boldsymbol{\cdot} \boldsymbol{s}^{\boldsymbol{\pi}}_{\boldsymbol{\tau+1}}) \right]$$

$$\frac{\partial F}{\partial s^{\pi}_{\tau}} = 0 \quad \Rightarrow \; ln(\boldsymbol{s}^{\boldsymbol{\pi}}_{\boldsymbol{\tau}}) = ln\,(\boldsymbol{B}^{\boldsymbol{\pi}}_{\boldsymbol{\tau-1}})\boldsymbol{s}^{\boldsymbol{\pi}}_{\boldsymbol{\tau-1}} - ln\,(\boldsymbol{A}) \cdot \boldsymbol{o}^{\pi}_{\tau} + ln(\boldsymbol{B}^{\boldsymbol{\pi}}_{\boldsymbol{\tau}}) \cdot \boldsymbol{s}^{\boldsymbol{\pi}}_{\boldsymbol{\tau+1}})$$

Using the softmax function to normalise the probability distribution, the equation for the updated probability distribution over states is:

$$\boldsymbol{s}^{\boldsymbol{\pi}}_{\boldsymbol{\tau}} = \sigma \left(ln\,(\boldsymbol{B}^{\boldsymbol{\pi}}_{\boldsymbol{\tau-1}})\boldsymbol{s}^{\boldsymbol{\pi}}_{\boldsymbol{\tau-1}} - ln\,(\boldsymbol{A}) \cdot \boldsymbol{o}^{\pi}_{\tau} + ln\,(\boldsymbol{B}^{\boldsymbol{\pi}}_{\boldsymbol{\tau}}) \cdot \boldsymbol{s}^{\boldsymbol{\pi}}_{\boldsymbol{\tau+1}}) \right)$$

Upon receiving a new observation, the agent immeadiately updates estimates for s^{π}_{τ} (and calculates $\boldsymbol{F}_{\boldsymbol{\pi}}$ in the process) by iterating the update above until convergence. This is an update over a fast timescale, as estimates of $\boldsymbol{s}^{\boldsymbol{\pi}}_{\boldsymbol{\tau}}$ are updated with each iteration immeadiately each observation occurs.

3.3 The expected free energy of policies in the future $G(\pi)$

As noted above, $G(\pi)$ is the expected free energy over future time points if a policy π were pursued. It is effectively a path integral of free energy under policy π from just after the present time-point τ to the temporal horizon T, taking into account all possible sequences of states that could occur. The overall probability of a given policy depends on this quantity, and in order to derive update expressions for $\boldsymbol{\pi}$ it will be necessary to derive a compact expression for $G(\pi)$. Let t represent the present time, then, using equation (1):

$$G(\pi) = \sum_{\tau=t+1}^{T} G(\pi, \tau)\,,$$

$$-G(\pi, \tau) = E_{Q(o_\tau, s_\tau \mid \pi)}\,[ln\,P(o_\tau, s_\tau \mid \pi) - ln\,Q(s_\tau \mid \pi)] \tag{8}$$

It is important to note that the agent must calculate $G(\pi, \tau)$ using its beliefs at t (the present). For each future time-point it uses $Q(o_\tau, s_\tau \mid \pi)$ to calculate the expected values in (8). $Q(o_\tau, s_\tau \mid \pi)$ are the agent's beliefs about distributions over future states based on its beliefs in the present. Under the generative model $P(o_\tau, s_\tau \mid \pi) = Q(s_\tau \mid o_\tau, \pi)\,P(o_\tau)$ ([4]):

$$\begin{aligned}
-G(\pi, \tau) &= E_{Q(o_\tau, s_\tau \mid \pi)}\,[ln\,P(o_\tau, s_\tau \mid \pi) - ln\,Q(s_\tau \mid \pi)] \\
&= E_{Q(o_\tau, s_\tau \mid \pi)}\,[ln\,Q(s_\tau \mid o_\tau, \pi) + ln\,P(o_\tau) - ln\,Q(s_\tau \mid \pi)] \\
&= E_{Q(o_\tau, s_\tau \mid \pi)} \left[ln \frac{Q(s_\tau \mid o_\tau, \pi)}{Q(s_\tau \mid \pi)} + ln\,P(o_\tau) \right] \\
&= E_{Q(o_\tau, s_\tau \mid \pi)} \left[ln \frac{Q(o_\tau \mid s_\tau, \pi)}{Q(o_\tau \mid \pi)} + ln\,P(o_\tau) \right] \qquad (*) \\
&= E_{Q(o_\tau, s_\tau \mid \pi)} \left[ln \frac{P(o_\tau)}{Q(o_\tau \mid \pi)} + ln\,Q(o_\tau \mid s_\tau, \pi) \right]
\end{aligned}$$

Using $Q(o_\tau \mid s_{\tau,}, \pi) = P(o_\tau \mid s_{\tau,})$ (since predicted outcomes are exactly represented in $\boldsymbol{A}$ and independent of policy, given s_τ):

$$G(\pi,\tau) = E_{Q(o_\tau,|\pi)}\left[ln\frac{Q(o_\tau \mid \pi)}{P(o_\tau)}\right] + E_{Q(s_\tau|\pi)}H\left[P(o_\tau \mid s_{\tau,})\right].$$
$$= D_{KL}\left[Q(o_\tau \mid \pi) \parallel P(o_\tau)\right] + E_{Q(s_\tau|\pi)}H\left[P(o_\tau \mid s_{\tau,})\right]$$

Where H represents the entropy of the distribution $P(o_\tau \mid s_{\tau,})$. In terms of the agent's beliefs, the above expression becomes:

$$G(\pi,\tau) = \boldsymbol{o}_{\boldsymbol{\tau}}^{\boldsymbol{\pi}} \cdot \left[ln\, \boldsymbol{o}_{\boldsymbol{\tau}}^{\boldsymbol{\pi}} - \boldsymbol{C}\right] + \boldsymbol{s}_\tau^\pi \cdot \boldsymbol{H}. \tag{9}$$

Where $\boldsymbol{H}$ is the vector consisting of the diagonal elements of $-\boldsymbol{A}\, ln(\boldsymbol{A})$, and the agent's priors over outcomes, $P(o_\tau)$, are associated explicitly with utility and are denoted simply by the vector $\boldsymbol{C}$. Note if the expression marked (*) is rearranged, then $G(\pi,\tau)$ can be written

$$G(\pi,\tau) = \underset{(-ve)\ epistemic\ value}{E_{Q(o_\tau,s_\tau|\pi)}\left[ln\, Q(o_\tau \mid \pi) - ln\, Q(o_\tau \mid s_\tau, \pi)\right]} - \underset{utility}{E_{Q(o_\tau,s_\tau|\pi)}\left[lnP(o_\tau)\right]}$$

Because policy probability depends on estimates of $G(\pi,\tau)$, this indicates action selection is driven in tandem by both utility and epistemic value, that is, the reduction of uncertainty [3].

3.4 Update equations for policies (π)

Equation (6) can now be written

$$F = D_{KL}\left[Q(\pi) \parallel P(\pi)\right] + D_{KL}\left[Q(\boldsymbol{A},\boldsymbol{B},\boldsymbol{D},\gamma) \parallel P(\boldsymbol{A},\boldsymbol{B},\boldsymbol{D},\gamma)\right] + \boldsymbol{\pi} \cdot \boldsymbol{F}_{\boldsymbol{\pi}} \tag{10}$$

$$Term\,1 = E_{Q(x)}\left[lnQ(\pi) - ln\left[\sigma(-\gamma.\cdot \boldsymbol{G}_\pi)\right]\right] = \boldsymbol{\pi} \cdot (ln\,\boldsymbol{\pi} + \gamma \boldsymbol{G}_\pi)) - E_Q(lnZ)$$

$$Term\,2 = E_{Q(x)}\left[\frac{lnQ(\boldsymbol{A})}{lnP(A)} + \frac{lnQ(\boldsymbol{B})}{lnP(B)} + \frac{lnQ(\boldsymbol{D})}{lnP(D)} + \frac{lnQ(\boldsymbol{\gamma})}{lnP(\gamma)}\right]$$

Where $\boldsymbol{F}_{\boldsymbol{\pi}} = (F(\pi_1), F(\pi_2)...)$, $\boldsymbol{G}_{\boldsymbol{\pi}} = (G(\pi_1), G(\pi_2)...)$ and $Z = \sum_i e^{-\gamma \cdot G_{\pi_i}}$, the denominator of the softmax function σ.

The update equations for policy probabilities can now be easily derived:

$$\frac{\partial F}{\partial \boldsymbol{\pi}} = ln\,\boldsymbol{\pi} + \gamma \boldsymbol{G}_{\boldsymbol{\pi}} + \boldsymbol{F}_{\boldsymbol{\pi}};$$

$$\frac{\partial F}{\partial \boldsymbol{\pi}} = 0 \;\Rightarrow ln\,\boldsymbol{\pi} = -\gamma \boldsymbol{G}_{\boldsymbol{\pi}} - \boldsymbol{F}_{\boldsymbol{\pi}}$$

$$\boldsymbol{\pi} = \sigma\left(-\gamma \boldsymbol{G}_{\boldsymbol{\pi}} - \boldsymbol{F}_{\boldsymbol{\pi}}\right)$$

3.5 Update equations for precision (γ)

Using the results in section 7 and disregarding constants, the expression in term 2 of the free energy involving γ can be written:

$$E_{Q(x)}\left[\frac{lnQ(\boldsymbol{\gamma})}{lnP(\gamma)}\right] = E_{Q(x)}\left[ln(\boldsymbol{\beta}) - \boldsymbol{\beta\gamma}\right] - E_{Q(x)}\left[ln(\beta) - \beta\boldsymbol{\gamma}\right] = -ln\boldsymbol{\gamma} - 1 + \beta\boldsymbol{\gamma}\,,$$

$$\text{using } \beta = \frac{1}{E_{Q(x)}(\gamma)},\quad ln\beta = -lnE_{Q(x)}(\gamma) = -ln\boldsymbol{\gamma}.$$

In the above, terms of the form $E_{Q(x)}[P(parameter)]$ are values for variables in P (such as $\boldsymbol{A}$ or γ) based on the agent's beliefs Q about the expected value of parameters (such as $\boldsymbol{a}$ or $\boldsymbol{\beta}$). Expected values under beliefs in Q for individual parameters such as β in P are meaningless and are discounted. Dropping constants:

$$E_{Q(x)}\left[\frac{lnQ(\gamma)}{lnP(\gamma)}\right] = -ln(\boldsymbol{\gamma}) + \boldsymbol{\beta\gamma}$$

Using this, and remembering that the free energy also has a dependence on γ via term 1, the update equation for γ is

$$\begin{aligned}\frac{\partial F}{\partial \gamma} &= -\frac{1}{\boldsymbol{\gamma}} + \beta + \boldsymbol{\pi}\cdot\boldsymbol{G_\pi} + \frac{\partial}{\partial\gamma}E_Q(ln\sum_i e^{-\gamma G_{\pi_i}})\\ &= \boldsymbol{\pi}\cdot\boldsymbol{G_\pi} - \boldsymbol{\beta} + \beta + \frac{1}{\sum_i e^{-\gamma\cdot G(\pi_i)}}\sum_i G(\pi_i)\,e^{-\gamma G\pi_i}\\ &= \beta - \boldsymbol{\beta} + \left[\boldsymbol{\pi} - \sigma(-\gamma\boldsymbol{G_\pi})\right].\boldsymbol{G_\pi}\end{aligned}$$

$$\frac{\partial F}{\partial\gamma} = 0 \quad\Rightarrow \boldsymbol{\beta} = \beta + \left[\boldsymbol{\pi} - \boldsymbol{\pi_0}\right].\boldsymbol{G_\pi}$$

where $\boldsymbol{\pi_0} = \sigma(-\gamma\boldsymbol{G_\pi})$. The updates for precision and policies are iterated to convergence. This takes place after the fast updates to states have occured - meaning that $\boldsymbol{\pi}$ and γ are updated on a slower timescale relative to the preceeding observation.

4 Bayesian Model Average, state action prediction errors and action selection

At each time during the trial, the agent uses the updated distribution over states and policy probabilities to form the Bayesian Model Average (BMA) over states. This is an 'overall' probability distribution for states over time, taking into account the probability of the various policies (which predict different states at each time):

$$\boldsymbol{S_\tau} = \sum_p \boldsymbol{\pi}_p \cdot \boldsymbol{s}_\tau^{\pi_p}$$

Hereπ_p is the probability of policy p, whilst $s_\tau^{\pi_p}$is the probability of state s under policy p at time τ. In this paper we have introduced the *state-action prediction error* as a measure of the change to the BMA over states

between time-points. If the agent's beliefs about the environment are accurate, this should change smoothly as the agent moves through time. Exceptions occur when lower probability events occur requiring an abrupt update to action planning (and to predicted states), or when the environment is no longer well described by the agent's beliefs.

$$SAPE = \sum_{\tau} D_{KL}(S_{\tau}^{t} \parallel S_{\tau}^{t-1})$$

Where s_t^{τ} is the BMA distribution over states for the time-point τ within the task, whilst t is the time at which the BMA is calculated.

4.1 Action Selection

Finally, the BMA is used to select an action. The agent tries to select an action at time t that will bring about the outcome predicted by the BMA over states at time $t+1$:

$$u \underset{min(u)}{=} D_{KL}(\boldsymbol{A}\boldsymbol{S_{t+1}} \parallel \boldsymbol{A}\,(\boldsymbol{B}(u)\boldsymbol{S}_t))$$

This action is used to generate a new observation, which triggers a new round of updates on states, policy probabilities and precision, continuing the cycle of inference and action until the end of the trial.

5 Update equations for concentration parameters

The update equations for the concentration parameters $\boldsymbol{a}, \boldsymbol{b}$ and $\boldsymbol{d}$ are derived in exactly the same way as for the other parameters above. The resulting expressions make use of the agent's experiences - its full history of states and outcomes - over the entire trial. This means that the concentration parameters are updated at the end of each trial: the slowest of the three timescales presented.

As above, the terms in the expression for F which depend on $\boldsymbol{a}, \boldsymbol{b}$ and $\boldsymbol{d}$ are first re-written in terms of model parameters. For $\boldsymbol{d}$, again using results in section 7 and dropping constants :

$$E_{Q(x)}\left[\frac{lnQ(\boldsymbol{D})}{lnP(D)}\right] = (\boldsymbol{d} - d)\cdot ln\,\boldsymbol{D} - lnB(\boldsymbol{d}),$$

$$since\;\; Q(\boldsymbol{D}) = \frac{1}{B(\boldsymbol{d})}\prod_{i=1}^{k} D_i^{d_i - 1};\; and\; ln(Q(\boldsymbol{D}) = \sum_i (\boldsymbol{d}_i - 1)\,lnD_i - lnB(\boldsymbol{d})\,,$$

We also note that $\boldsymbol{\pi}\cdot\boldsymbol{F_{\pi}}$also has a dependence on $\boldsymbol{D}$ via $F(\pi, \tau = 1)$ (equations 7 and 11).

For the A and B matrices there is a similar expression for each column (indexed by j below). These are then summed:

$$E_{Q(x)}\left[\frac{lnQ(\boldsymbol{A})}{lnP(A)}\right] = \sum_j (\boldsymbol{a_j} - a_j)\cdot ln\,\boldsymbol{A_j} - lnB(\boldsymbol{a_j})$$

$$E_{Q(x)}\left[\frac{lnQ(\boldsymbol{B})}{lnP(B)}\right] = \sum_j (\boldsymbol{b_j} - b_j)\cdot ln\,\boldsymbol{B_j} - lnB(\boldsymbol{b_j})$$

N.B. there is a separate $\boldsymbol{B}$ matrix - and corresponding term in the free energy - for each action u.
The update equation for the concentration parameters $\boldsymbol{d}$ can now be derived. Using results from section 7:

$$\frac{\partial F}{\partial \boldsymbol{d}} = \frac{\partial}{\partial d}\left[(\boldsymbol{d} - d)\cdot ln\ \boldsymbol{D} - lnB(\boldsymbol{d}) + \boldsymbol{\pi}\cdot \boldsymbol{F_\pi}(\tau = 1)\right]$$
$$= \frac{\partial ln\boldsymbol{D}}{\partial \boldsymbol{d}}\left[(\boldsymbol{d} - d) + ln\ \boldsymbol{D} - ln\boldsymbol{D} - \boldsymbol{s}_1\right]$$

$$\frac{\partial F}{\partial \boldsymbol{d}} = 0 \quad \Rightarrow \boldsymbol{d} = d + \boldsymbol{s}_1$$

Where $\boldsymbol{s_1} = \sum_p \boldsymbol{\pi}_p \cdot \boldsymbol{s}_1^{\pi_p}$, the Bayesian Model Average for the state at $\tau = 1$ (i.e. the average prediction for the state at $\tau = 1$, taking into account the probability of each policy - see below). For concentration parameters $\boldsymbol{a}$:

$$\frac{\partial F}{\partial \boldsymbol{a}} = \frac{\partial}{\partial \boldsymbol{a}}\sum_j\left[(\boldsymbol{a_j} - a_j)\cdot ln\ \boldsymbol{A_j} - lnB(\boldsymbol{a_j})\right] + \frac{\partial}{\partial \boldsymbol{a}}\boldsymbol{\pi}\cdot\sum_{\tau=1}^{T}\boldsymbol{s_\tau^\pi}\cdot\left[-ln(\boldsymbol{A})\cdot\boldsymbol{o}_\tau\right]$$
$$= \frac{\partial ln(A)}{\partial \boldsymbol{a}}\left[(\boldsymbol{a} - a) - \sum_\tau(\boldsymbol{o_\tau}\otimes\boldsymbol{s_\tau})\right]$$

$$\frac{\partial F}{\partial \boldsymbol{a}} = 0 \quad \Rightarrow \boldsymbol{a} = a + \sum_\tau(\boldsymbol{o_\tau}\otimes\boldsymbol{s_\tau}) \ \ and \ \ \boldsymbol{s_\tau} = \sum_p \boldsymbol{\pi}_p\cdot\boldsymbol{s}_\tau^{\pi_p}$$

Where p indexes policies, and $\boldsymbol{s_\tau}$ is the Bayesian Model Average over states. Finally, for each $\boldsymbol{b}$ matrix

$$\frac{\partial F}{\partial \boldsymbol{b(u)}} = \frac{\partial}{\partial \boldsymbol{b(u)}}\sum_j\left[(\boldsymbol{b(u)_j} - b(u)_j)\cdot ln\ \boldsymbol{B(u)_j} - lnB(\boldsymbol{b(u)_j})\right] + \frac{\partial}{\partial \boldsymbol{b(u)}}\boldsymbol{\pi}\cdot\sum_{\tau=1}^{T}\boldsymbol{s_\tau^\pi}\cdot ln\left(\boldsymbol{B(u)_{\tau-1}^\pi}\right)\boldsymbol{s_{\tau-1}^\pi}$$
$$= \frac{\partial ln(\boldsymbol{B(u)})}{\partial b(u)}\left[(\boldsymbol{b(u)} - b(u)) - \sum_{\tau,\pi(\tau)=u}\pi_p\boldsymbol{s_\tau^{\pi_p}}\otimes\boldsymbol{s_{\tau-1}^{\pi_p}}\right]$$

Where policy $\pi = p$ predicts the action u at time τ.

$$\frac{\partial F}{\partial \boldsymbol{b(u)}} = 0 \quad \Rightarrow \boldsymbol{b(u)} = b(u) + \sum_{\tau,p(\tau)=u}\pi_p\,\boldsymbol{s_\tau^{\pi_p}}\otimes\boldsymbol{s_{\tau-1}^{\pi_p}}$$

6 Model decay

The maximum value of state action prediction error encountered during the trial is used as the input to a logistic function to calculate a 'decay factor' α:

$$\alpha = \alpha_{min} + \frac{\alpha_{max}}{1 + e^{k(SAPE-m)}}$$

In this paper, we have used $\alpha_{min} = 2$, $\alpha_{max} = 32$ throughout. We used a value k (the mean of the logistic) determined by the agent's initial learning of the task with a fixed value of α, enabling the agent to calibrate the

conversion between error and decay appropriately. In both tasks, the agent initially completed 100 trials with $\alpha = 16$. The mean of the logistic curve was then set one standard deviation higher than the mean value of SAPE encountered in the learning period. This gave a value of $k = 1.8$ for the explore/exploit task, and $k = 1$ for the Go/no-go task. Model decay is implemented alongside the updates for $\boldsymbol{a}, \boldsymbol{b}$ and $\boldsymbol{d}$ described above. For the $\boldsymbol{d}$ vector, the modified update takes the form

$$\boldsymbol{d} = d + S_1 - \frac{(d-1)}{\alpha}$$

Updates to the $\boldsymbol{b}$ and $\boldsymbol{a}$ distributions take the same form:

$$\boldsymbol{b}(u) = b(u) + \sum_{\tau, p(\tau)=u} \pi_p \, \boldsymbol{s_\tau^{\pi_p}} \otimes \boldsymbol{s_{\tau-1}^{\pi_p}} - \frac{(\boldsymbol{b}(\pi_p(\tau) = u) - 1)}{\alpha}$$

$$\boldsymbol{a} = a + \sum_{\tau} (\boldsymbol{o_\tau} \otimes \boldsymbol{S_\tau}) + \frac{a(o_\tau) - 1}{\alpha}$$

Where $a(o_\tau)$ are the same subset of matrix elements of a picked out by the outer product $\boldsymbol{o_\tau} \otimes \boldsymbol{s_\tau}$ (i.e. those in the row corresponding to the observation o_τ). In the update equation for $\boldsymbol{b}$ the same principle holds, i.e. the decay affects the subset of elements picked out by the outer product $\boldsymbol{s_\tau^{\pi_p}} \otimes \boldsymbol{s_{\tau-1}^{\pi_p}}$. The update equations above are implemented only for non-zero elements of $\boldsymbol{a}, \boldsymbol{b}$ and $\boldsymbol{d}$ (i.e. only for transitions between states / outcomes that are in any way possible in the task being modelled).

7 Notes: useful results for Dirichlet, beta and gamma distributions

7.0.1 Dirichlet distributions, gamma and beta functions

Dirichlet distributions are used throughout the derivation above to provide probability distributions over categories (for instance, these distributions are used for the vector $\boldsymbol{D}$ and columns of the $\boldsymbol{A}$ and $\boldsymbol{B}$ matrices). The following results are used:

The probabilities in the vector $\boldsymbol{D}$ are given by a Dirichlet distribution parameterised by concentration parameters $\boldsymbol{d}$ such that

$$Dir(D_1, D_1 .... D_k;\, d_1,\, d_2 ...\, d_k) = \frac{1}{B(\boldsymbol{d})} \prod_{i=1}^{k} D_i^{d_i - 1},$$

where B is the multivariate beta function. The expected values of D and of $ln\,(D)$ are given by

$$E\,[D_i] = \frac{d_i}{\sum_k d_k} \;, \qquad E\,[ln(D_i)] = \psi(d_i) - \psi(\sum_k d_k).$$

Where ψ represents the digamma function. In the $\boldsymbol{A}$ and $\boldsymbol{B}$ matrices, each column is a Dirichlet distribution such that

$$E\,[A_{ij}] = \frac{a_{ij}}{\sum_i d_{ij}} \;, \qquad E\,[ln(A_{ij})] = \psi(a_i) - \psi(\sum_i a_{ij})$$

The following result for the derivatives of the gamma and beta functions are also used in the derivation above:

$$B(\boldsymbol{d}) = \frac{\prod_i^k \Gamma(d_i)}{\Gamma(\sum_i^k d_i)} \;, \quad \Gamma'(d_i) = \Gamma(d_i)\psi(d_i)$$

$$\frac{\partial B(\boldsymbol{d})}{\partial d_i} = B(\boldsymbol{d}) \left(\psi(d_i) - \psi(\sum_i^k d_i) \right) = B(\boldsymbol{d})\, E\left[ln(\boldsymbol{D_i})\right]$$

$$\frac{\partial\, lnB(\boldsymbol{d})}{\partial d_i} = E\left[ln(\boldsymbol{D_i})\right]$$

7.0.2 Gamma distributions

The parameter γ is described by a gamma distribution such that

$$P(\gamma\,;\,\alpha,\beta) = \frac{\beta^{\alpha}\,\gamma^{\alpha-1}\,e^{-\beta\gamma}}{\Gamma(\alpha)}$$

Where $\Gamma(\alpha)$ is the gamma function. For simplicity,$\alpha = 1$ in the derivations above. The following results are used:

$$E\left[\gamma\right] = \frac{1}{\beta}\ , \qquad ln(P(\gamma)) = ln(\beta) - \beta\gamma$$

References

[1] Charles W. Fox and Stephen J. Roberts. A tutorial on variational Bayesian inference. *Artificial Intelligence Review*, 38(2):85–95, aug 2012.

[2] Karl Friston. The free-energy principle: a rough guide to the brain? *Trends in Cognitive Sciences*, 13(7):293–301, 2009.

[3] Karl Friston, Thomas FitzGerald, Francesco Rigoli, Philipp Schwartenbeck, and Giovanni Pezzulo. Active Inference: A Process Theory. *Neural Computation*, 29(1):1–49, jan 2017.

[4] Karl Friston, Francesco Rigoli, Dimitri Ognibene, Christoph Mathys, Thomas Fitzgerald, and Giovanni Pezzulo. Cognitive Neuroscience Active inference and epistemic value. *Cognitive Neuroscience*, 2015.

[5] David J.C. MacKay. Information Theory, Inference & Learning Algorithms. chapter 33. Cambridge University Press, New York, NY, US, 2002.
