# Supplementary material for: Locus Coeruleus tracking of prediction errors optimises cognitive flexibility: An Active Inference model
Source: PLoS Comput Biol. 2019 Jan 4;15(1):e1006267. doi: 10.1371/journal.pcbi.1006267 (PMC6334975; doi:10.1371/journal.pcbi.1006267)
Supplement: S2 Appendix — (DOCX) [file pcbi.1006267.s002.docx]

**Pseudocode**

The MATLAB code for the simulations described in this paper are available at <https://github.com/AnnaCSales/ActiveInference>. Below we provide a ‘pseudocode’ overview of how scheme described in Boxes 1, 2 and Appendix 1 are implemented.

**Function Go_NoGo or ExploreExploit [***this is the script that defines the task]*

Define agent’s initial values of a, b, d, A, B, C, D, U, V ,ɣ

Define true environmental matrices A_ENV, B_ENV

Define true starting state s and the first observation o

Define number of trials n_t, and the number of timepoints in each trial T

Define the number of iterations for update equations

Define an ‘MDP’ object for each trial to hold all parameters, actions and inferences

Run the MDP through the Active Inference engine by calling spm_MDP(input = MDP objects)

**end**

**function spm_MDP( takes as input MDP object )**

for i=1 to number of MDP objects

if i>1

give current (i^th^) MDP the values (**a,b,c,d,**β, o) taken from the end of the previous (i-1^th^) MDP

else

take the initial values for (**a,b,c,d,**β,o) defined in the Task Script.

end

pass the current MDP to the next part of the script

end

Calculate expected values of **A,B,D, ln(A), ln(B), ln(D)** from **a,b,d** parameters

for t=1 to T  *[Calculate results for each time t=1,2…T in the trial for this MDP]*

for p=1 to number of policies

Iterate Ni times {

for j=1 to T  *[move forward and backward in time updating state probabilities]*

Calculate $s_{t=j}^{\pi=p}$ using the update equation for states

Calculate free energy of individual policy p at time j, $F(\pi,\tau)$

end

}

end

for p=1 to number of policies

Calculate expected free energy of policy in the future $G(\pi,\tau)$

end

Iterate Ni times {

Calculate $\boldsymbol{\pi}$and $\gamma$ using their update equations

}

Calculate Bayesian Model Average over states

if t>1 calculate State Action Prediction Error

Use Bayesian Model Average of states to select an action

Calculate next observation (for t+1) and next true state using environmental matrices / agent’s chosen action

end

Calculate α based on the maximum value of State Action Prediction Error observed

Use alpha to ‘decay’ **a,b,d** / increment **a,b**,**d** using update equations and the history of observations / states

Store all parameters, chosen actions, observations at each time point in the MDP.

**end**
