# Supplementary figures and images for: Locus Coeruleus tracking of prediction errors optimises cognitive flexibility: An Active Inference model
Source: PLoS Comput Biol. 2019 Jan 4;15(1):e1006267. doi: 10.1371/journal.pcbi.1006267 (PMC6334975; doi:10.1371/journal.pcbi.1006267)

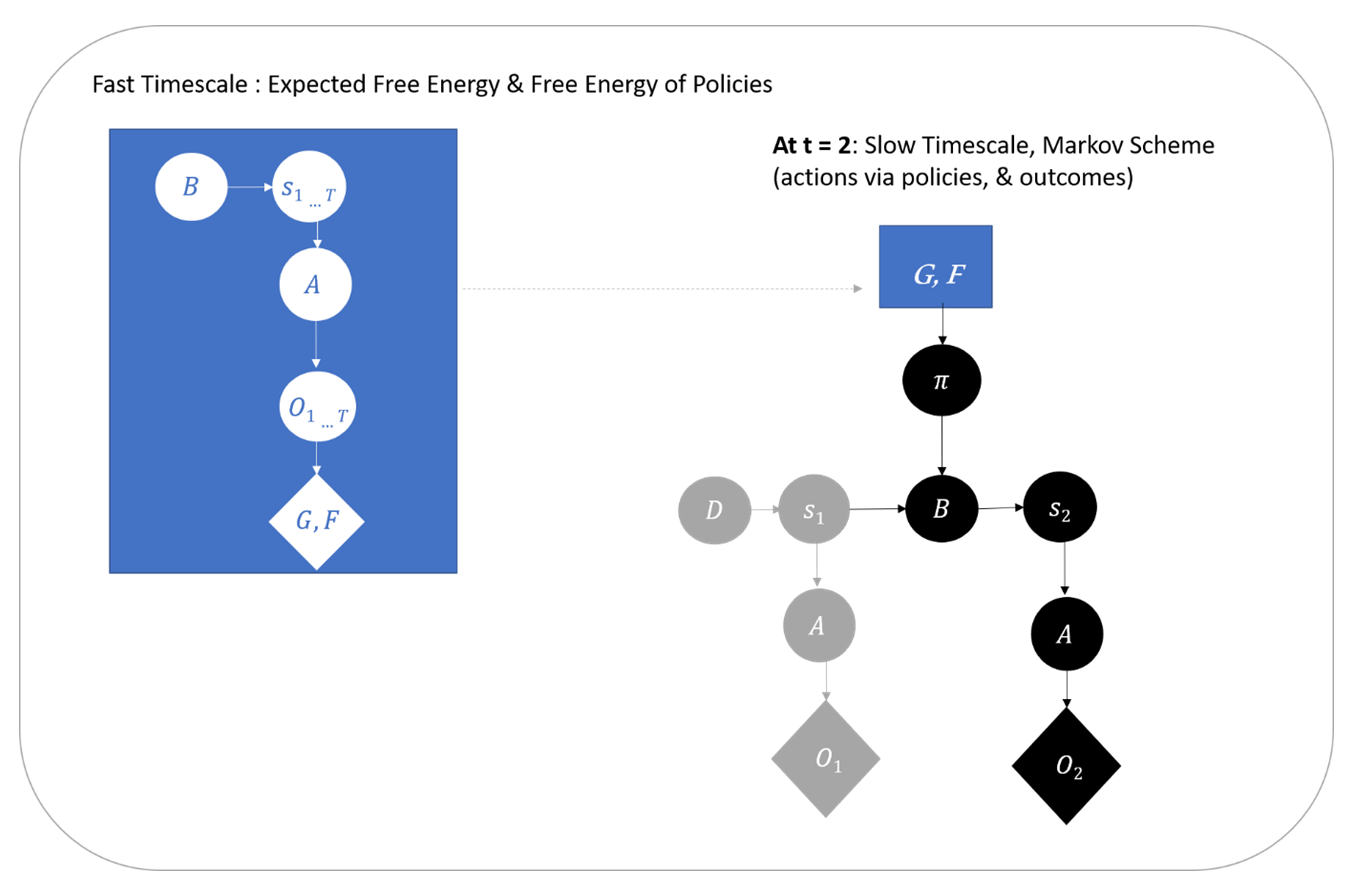

Supplement: S1 Fig — First the free energy and expected free energy of policies is evaluated. Next these serve as priors on the probability of a policy, which in turn leads to a selected action, state transitions and outcomes. In Appendix 1 we provide a full description of the model. (TIF) [file pcbi.1006267.s003.tif]

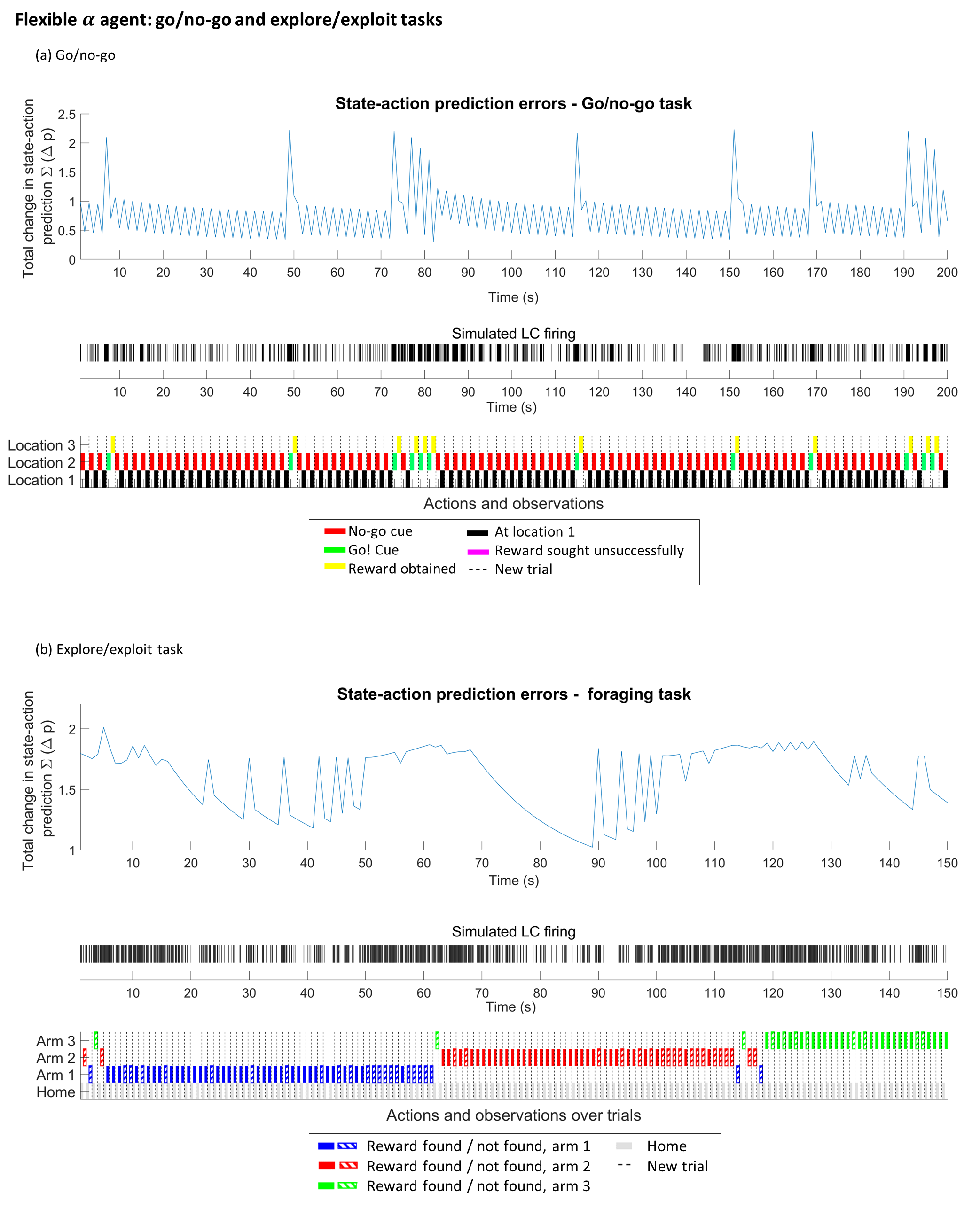

Supplement: S2 Fig — (a) shows the performance of the agent in the go/no-go task. The prediction error output is similar in form to the output from the task played by the agent with α = 16 in Fig 5, except for the more pronounced reduction in the size of the state-action prediction error peaks in response to consecutive cues. (b) shows the performance during the explore/exploit task with the same parameters as in Fig 7(A) and 7(B), that is, a with changes in the location of the high probability arm (p = 0.7) every 50 trials. (TIF) [file pcbi.1006267.s004.tif]

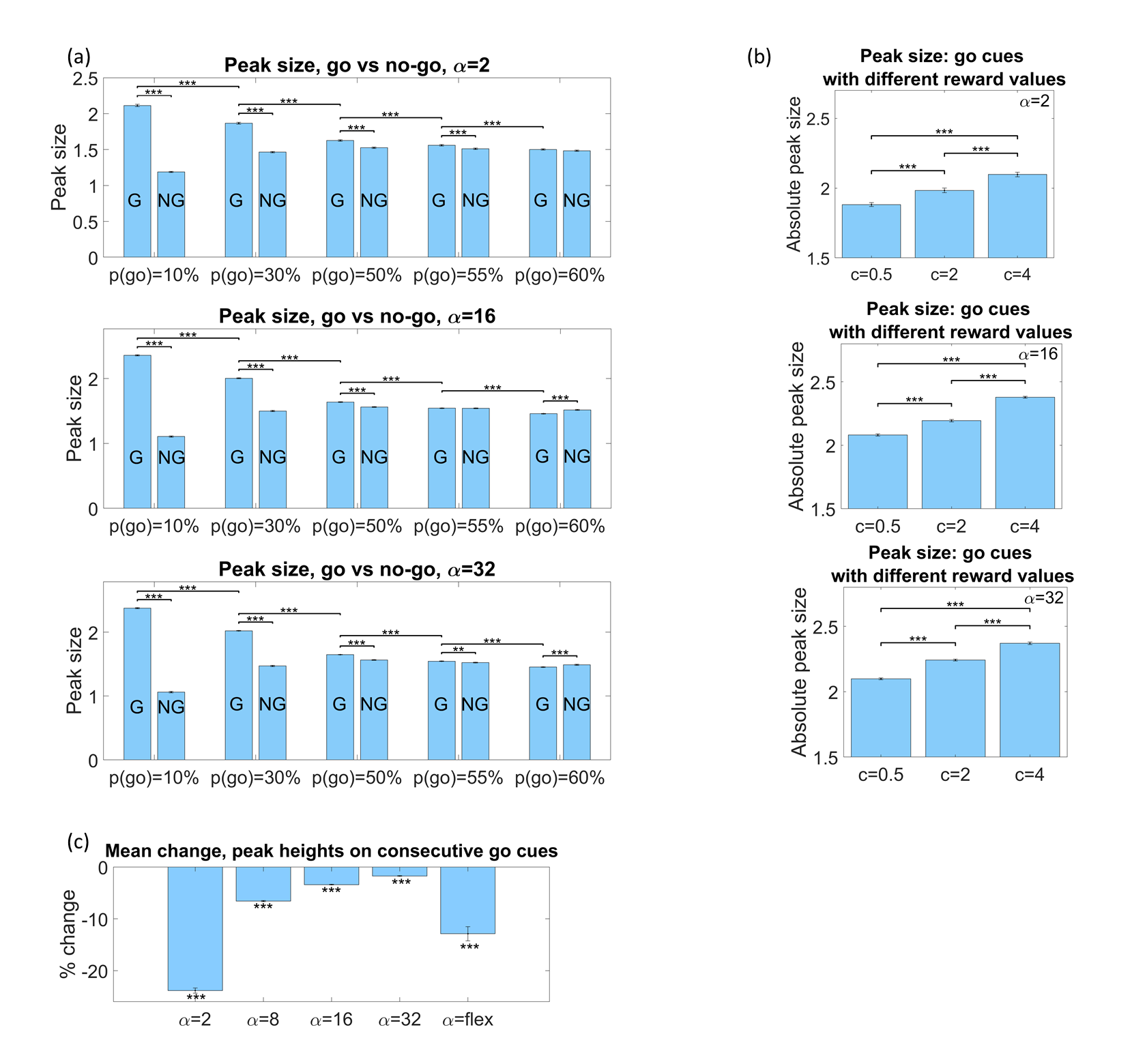

Supplement: S3 Fig — All plots show averages over 2000 trials. (a) shows the changes in prediction error peak responses during either ‘go’ (marked ‘G’) cues or ‘no-go’ (marked ‘NG’) cues. All agents display a larger ‘go’ response for rarer cues. Additionally, responses for ‘go’ cues are consistently larger than those for ‘no-go’ cues when the ‘go’ is more probable than, or equally probable as, the ‘no-go’ cue. This effect persists up to a ‘go’ probability of 55%. When the probability of the cue is increased further, the peaks are equal in size or reversed. (b) shows the effect of changing the reward size. As in Fig 8, all agents display a larger state-action prediction error response when the reward is larger. (c) shows the reduction in peak size caused by presenting ‘go’ cues consecutively. This reduction is greater for the more flexible (lower α) agents, reflecting the larger changes to the agent’s model caused by the consecutive ‘go’ cues. (TIF) [file pcbi.1006267.s005.tif]
